# HIV in women

**DOI:** 10.7448/IAS.15.6.18075

**Published:** 2012-11-11

**Authors:** F Mulcahy

**Affiliations:** St James's Hospital, Dublin, Ireland

## Abstract

Globally over 50% of HIV-infected individuals are women. With the widespread use of HAART, we can expect women to have mortality rates approaching normal. Indeed, studies have shown that women may expect a slower disease progression than men following seroconversion; furthermore, it appears that female who injects drugs can live longer than their male counterparts. However, other studies from cohort analysis have reported worse outcomes in women. In essence, many studies are consistently underpowered to adequately address these questions. The proportion of women in clinical trials remains at 20 to 30%, with pregnancy potential being a major exclusion factor. Hence, many questions remain unanswered. Recent data suggest women are more likely to present late with a new AIDS diagnosis. Why this should be the case is not well understood. In addition, HIV-positive women should have the same access to reproduction health as their negative counterparts, but unfortunately many inequalities remain. Advise on contraception and fertility services are very variable across both the developed and developing world. Data are limited on the most appropriate use of contraceptives in the presence of HAART, the possible drug interactions and possible increased risk of HIV transmission. There remain significant differences in guidelines regarding prevention of mother-to-child transmission (MTCT) across Europe, and implications of stopping and starting HAART for MTCT have not been adequately addressed. The mode of timing of delivery, and the effect of length of time of ruptured membranes on this decision is also contentious. Further issues relate to the desire for HIV-positive women to breastfeed in the setting of HIV viral suppression, where some guidelines now support women in this situation and others categorically would inform child protection authorities. Finally as women age it is more difficult to separate the effect of the menopause and its symptoms from the increased HIV-related cardiovascular and bone fracture risk. This presentation aims to discuss key issues which conflicting or inadequate data fail to resolve.

